# The anti-cyclic citrullinated peptide response in tuberculosis patients is not citrulline-dependent and sensitive to treatment

**DOI:** 10.1186/ar2913

**Published:** 2010-01-25

**Authors:** Ori Elkayam, Refael Segal, Daniele Bendayan, Robert van Uitert, Carla Onnekink, Ger JM Pruijn

**Affiliations:** 1 Department of Internal Medicine F and the Department of Rheumatology, Tel Aviv Medical Center and The "Sackler" Faculty of Medicine, Tel Aviv University, 6 Weizman Street, Tel Aviv 64239, Israel; 2 Department of Geriatrics, Shmuel-Harofeh Geriatric Medical Centre and the "Sackler" Faculty of Medicine, University of Tel Aviv, POB 2, Beer Yaakov 70300, Israel; 3 Department of Tuberculosis, Shmuel-Harofeh Geriatric Medical Center and the "Sackler" Faculty of Medicine, University of Tel Aviv, POB 2, Beer Yaakov 70300, Israel; 4 Euro-Diagnostica AB, Beijerinckweg 18, NL-6827 BN, Arnhem, The Netherlands; 5 Department of Biomolecular Chemistry, Nijmegen Center for Molecular Life Sciences, Institute for Molecules and Materials, Radboud University Nijmegen, NL-6500 HB Nijmegen, The Netherlands

## Abstract

**Introduction:**

Patients with tuberculosis (TB) frequently produce anti-citrullinated protein antibodies (ACPA). The objective of this study is to characterize the citrulline-dependence of the ACPA reactivity in sera of patients with mycobacterium infections.

**Methods:**

Serum samples of 134 patients with untreated mycobacterium infections (122 TB, 12 nontuberculous mycobacterium) were tested for antibodies against both the citrullinated (Cit) and the non-citrullinated (Arg) form of 2 cyclic synthetic peptides. In 33 patients, a follow-up sample was tested six months after starting anti-mycobacterial drugs.

**Results:**

A substantial proportion of patients with mycobacterial infections demonstrated antibodies against 0401Cit, 0401Arg, 0722Cit and 0722Arg. Fourteen patients demonstrated anti-0401Cit, 83 anti-0401Arg, 22 anti-0722Cit and 61 anti-0722Arg, while none of these antibodies were detected in the 20 healthy controls. All the patients but one, who were anti-0401Cit and anti-0722Cit positive, demonstrated reactivity against the respective Arg peptide. In the subset of 33 patients with a follow-up test six months after starting treatment, the mean levels of antibodies to 0401Cit, 0401Arg, 0722Cit and 0722Arg significantly decreased after treatment. All the patients who were anti-0401Cit and anti-0722Cit positive turned negative after treatment. The presence of anti-0401Cit/Arg and anti-0722Cit/Arg was found to be significantly correlated with the presence of HIV.

**Conclusions:**

ACPA may be found in patients with TB. In most of the cases, the reactivity is citrulline independent. A positive cyclic citrullinated peptide (CCP) test in these patients should therefore be interpreted with care, and preferably followed by a control ELISA with a non-citrullinated antigen.

## Introduction

A group of autoantibodies, anti-citrullinated protein antibodies (ACPA), has been described in patients with rheumatoid arthritis (RA) [1]. The specificity for RA has been shown to be up to 98% in comparison with 0 to 1% of healthy controls and 2 to 5% of disease controls [1]. ACPA (most frequently detected by a cyclic citrullinated peptide, CCP, test) are present early in the disease process and may even predict the development of RA [2]. Schellekens et al. [3] and Girbal-Neuhauser et al. [4] have shown that ACPA specifically bind to substrates containing citrulline, a post-translationally modified amino acid. Citrullination, or peptidylarginine deimination, is the process by which the imino group of the guanidine moiety of arginine is hydrolysed, leading to the replacement of the protonated imino group by an oxygen atom [5]. When this occurs on an arginine present in a protein, the process is generally catalysed by a specific enzyme, the peptidylarginine deiminase (PAD).

It has recently been reported that anti-CCP2 antibodies can be detected in 9% of patients with type 1 autoimmune hepatitis (AIH-1) in the absence of recognizable rheumatoid arthritis overlap, and in some cases with high titres, comparable to those observed in RA [6]. However, it has been demonstrated that a high percentage of AIH-1 samples (42 to 50%) turned out to be reactive in a citrulline-independent manner [7].

We have reported increased levels of anti-CCP2 in up to 32% of patients with tuberculosis (TB) [8]. These patients also displayed increased frequency of other autoantibodies such as rheumatoid factor (RF), antinuclear antibodies and others [9]. Kakumanu and collaborators have recently reported that anti-CCP1 antibodies found in TB patients often react to the unmodified arginine-containing peptide as well [10].

The objective of our study was to characterize the observed ACPA reactivities in TB, especially regarding their dependence on the citrulline moiety, as is the case in RA, as well as their presence after treatment. For this purpose, we tested mycobacterial sera for reactivities with citrullinated peptides as well as the corresponding arginine-containing controls.

## Materials and methods

### Patients

One hundred and thirty-four consecutive patients with recently diagnosed active mycobacterial infections (122 with mycobacterium tuberculosis and 12 with nontuberculous mycobacterium (NTM)) participated in the study. All were admitted to the hospital department of tuberculosis, with clinical symptoms and radiological signs as well as positive cultures for *Mycobacterium*. A questionnaire was used to determine data on the clinical features of the disease, such as duration of symptoms, the presence of fever, cough, as well as rheumatological manifestations such as arthralgia/arthritis, myalgia, rash, mucocutaneous symptoms, sicca symptoms, spontaneous abortion, history of thrombosis, and familial history of autoimmune diseases. All the patients were tested for HIV, hepatitis B and C. Data on the resistance of the mycobacterium was collected. Serum samples were collected before starting treatment for TB or NTM infection in all patients and six months after starting treatment with anti-tuberculous drugs in 33 subjects. The samples were frozen at -20°C and subsequently tested.

### ELISA

Antibodies against both the citrullinated (Cit) and the non-citrullinated (Arg) form of 2 cyclic synthetic peptides (0401Cit, 0401Arg, 0722Cit and 0722Arg) were determined by ELISA. These peptides were synthesized with a C-terminal spacer and biotin tag. The 0401 peptides consist of 18 amino acids with the Cit/Arg at the sixth position, whereas the 0722 peptides consist of 13 amino acids with the Cit/Arg also at the sixth position. Cyclization was achieved by coupling the side chain of a cysteine residue at position 13 (of both peptide sets) to the amine at the N-terminus. Streptavidin-coated pre-blocked microtiter plates (StreptaWell, Roche-Applied-Science, Almere, The Netherlands) were coated with 10 μg/ml peptide diluted in PBS/0.1% BSA at room temperature for one hour. The coated plates were incubated with 100 μl/well serum for one hour (200 times diluted in PBS/1% BSA/0.05% Tween-20). This and the subsequent incubation step were performed at 37°C in a humidified atmosphere and were followed by washing steps with PBS/0.05% Tween-20. Antibodies were detected after incubation for one hour with 100 μl/well rabbit-anti-human IgG HRP-conjugated antibody (P0214, DAKO, Glostrup, Denmark) (1:10000 in PBS/1% BSA/0.05% Tween-20). Bound antibodies were visualized using either 100 μl/well of 3,3',5,5'-tetramethylbenzidine (TMB) solution mixed 1:1 with urea peroxide as a substrate, followed by 100 μl/well 2 M H_2_SO_4 _10 minutes later to stop the staining reaction. Optical density (OD) values were measured on an ELISA-reader at 450 nm.

A positive control consisting of an RA serum known to be positive for both citrullinated peptides, was included on each plate to determine similarity between plates coated with these peptides. Cut-off values to determine whether a peptide was recognized or not, were defined on the basis of the reactivity of sera from healthy control subjects. Cut-off values for the citrullinated and arginine-containing peptides were defined as the mean plus two times the standard deviation of the values of 10 control subjects, which were included on each plate.

The presence of anti-citrullinated protein antibodies in the TB sera was also monitored using the Immunoscan CCPlus kit (Euro-Diagnostica, Arnhem, The Netherlands) and the citrulline-dependency of these reactivities was determined in parallel by ELISA using plates coated with the corresponding arginine-containing peptides.

The study was approved by the ethics committee of the medical center. Appropriate informed consent was obtained from all patients, and the clinical research was conducted in accordance with guidelines for human experimentation specified by the Tel Aviv Sourasky Medical Center.

### Statistical analysis

Statistical analysis was performed by the SPSS software, using Student's t test and the chi square test to compare antibody titers or positivity rates, respectively, between TB patients and controls. The Pearson correlation coefficient was used for correlations between clinical and laboratory data. *P *< 0.05 was considered significant.

## Results

### Patients

Table 1 summarizes the clinical and demographic characteristics of 134 patients (122 TB and 12 NTM). One hundred and twenty-five patients suffered from pulmonary involvement (almost half of them demonstrating pulmonary cavitations on chest Xray) while 19 had extrapulmonary involvement. Twelve patients were HIV positive, nine hepatitis C and five hepatitis B surface antigen positive. Thirty-two patients were born in Israel while the rest originated from former Soviet Union (52), Africa (36) and Asia (14).

**Table 1:** Clinical and demographic characteristics of patients with mycobacterial infections

No. of patients	134
Age (range)	49 ± 20 ^a ^(17 to 99)
Gender; Male/Female (%)	69/31
**Origin **- (%)	
Israeli	32 (24%)
Russian	52 (39%)
African	36(27%)
Asian	14 (10%)
Length of symptoms in weeks (range)	17 ± 27 ^a ^(1 to 100)
Atypical mycobacterium	12 (9%)
Extrapulmonary TB	19 (14%)
Miliary disease	5 (4%)
Multidrug resistance	20 (15%)
HIV positive	12 (9%)
Hepatitis C positive	9 (7%)
Hepatitis B positive	5 (4%)

^a ^Mean ± SD

### Reactivity with citrullinated and corresponding arginine-containing peptides

**Figure 1:**
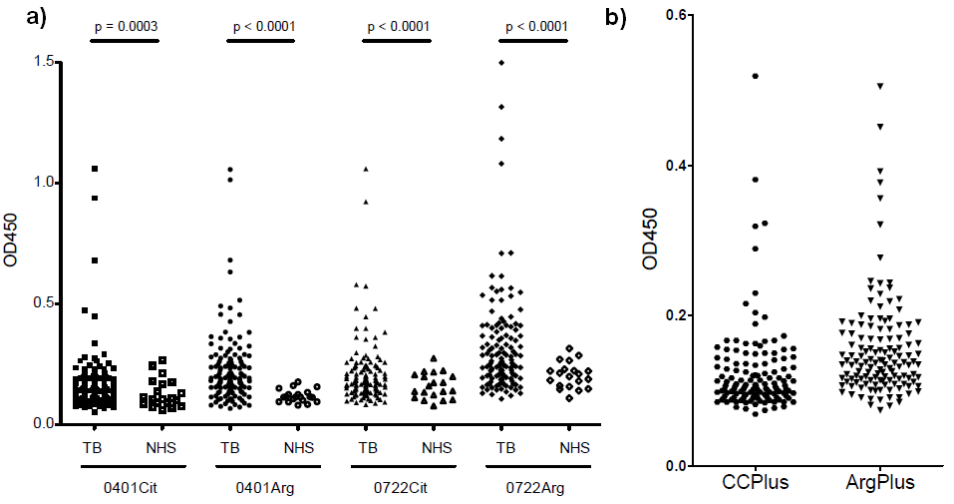
**Reactivity of TB and healthy control sera with citrullinated and corresponding arginine-containing peptides**. **a) **ELISA data obtained with peptides 0401Cit, 0401Arg, 0722Cit and 0722Arg. Within the group of patients with mycobacterial infections, 14 demonstrated anti-0401Cit, 83 anti-0401Arg, 22 anti-0722Cit and 61 anti-0722Arg while none of these antibodies were detected in the 20 healthy controls. **b) **ELISA data obtained with the CCPlus test and the corresponding arginine control (ArgPlus).

To investigate the ACPA reactivity in the sera of these TB and NTM patients, first two pairs of synthetic peptides were used. One of these, 0401Cit and 0401Arg, corresponds to the cfc1-cyc peptide used in the first generation CCP test (CCP1) and its arginine-containing equivalent cf0-cyc [11]; the sequence of the second pair, 0722Cit and 0722Arg, is related to a peptide used in the second generation CCP test (CCP2), which is defined in patent EP2071335 [12]. All of these peptides are cyclic and, in case of the citrullinated peptides, contain a single citrulline residue. A substantial proportion of patients with mycobacterial infections appeared to contain antibodies against 0401Cit, 0401Arg, 0722Cit and 0722Arg. Within the group of patients with mycobacterial infections, 14 patients demonstrated anti-0401Cit, 83 anti-0401Arg, 22 anti-0722Cit and 61 anti-0722Arg while none of these antibodies were detected in the 20 healthy controls (Figure 1A). All the patients but one, who were anti-0401Cit and anti-0722Cit positive, demonstrated reactivity against the respective Arg peptide (concordance of 92% for 0401 Cit/Arg and 95% for 0722Cit/Arg). These results indicate that the ACPA reactivities observed in TB patients are generally not citrulline-specific. Moreover, the frequency by which the non-citrullinated peptides are recognized by this patient group appeared to be higher than that of the citrullinated peptides.

The mean levels of Anti 0401 Cit/Arg and Anti 0722 Cit/Arg were significantly increased in patients with mycobacterial infections in comparison with healthy controls.

Next, ACPA reactivities in TB patient sera were determined by a commercially available CCP2 test (CCPlus). To investigate the citrulline-dependence of the antibodies detected by this test, the sera were analyzed in parallel with plates coated with the same peptides in which the citrulline residues were replaced by arginine residues (ArgPlus). The results of these analyses confirmed that ACPA are relatively frequently present in TB sera and that these reactivities are not citrulline-dependent (Figure 1B).

**Figure 2:**
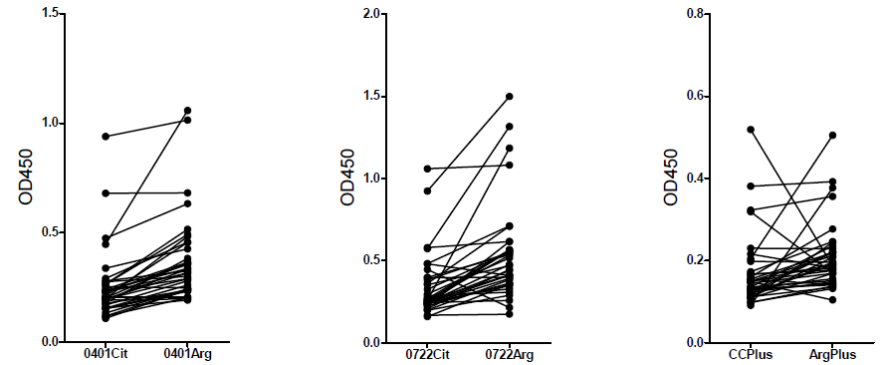
**Correlation between reactivities with citrullinated and with corresponding arginine-containing peptides**. The ELISA data for each pair of peptides are depicted for the 36 most highly reactive TB sera. Values for individual sera are connected by a line.

To demonstrate that individual patient sera are reactive with both the citrullinated and non-citrullinated peptides, the results for a selection of sera (the most highly reactive sera) are depicted in correlation diagrams in Figure 2. This indicates that most of the reactive TB sera indeed bind to these peptides irrespective of the presence of citrulline.

### Effect of anti-mycobacterial treatment on the reactivity with the 0401Cit, 0401Arg, 0722Cit and 0722Arg peptides

**Figure 3:**
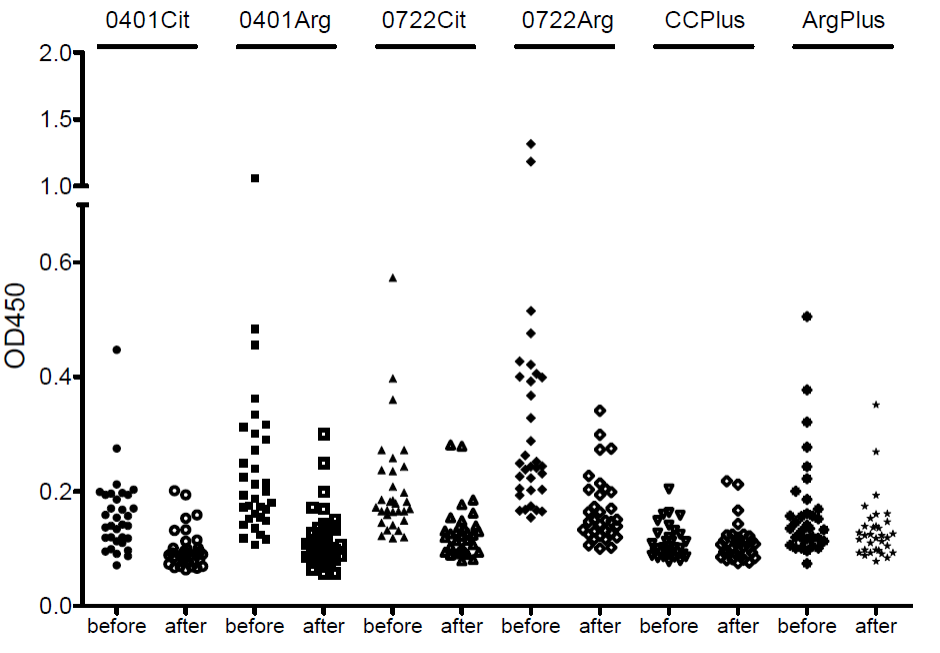
**Effect of anti-mycobacterial treatment on the reactivity with peptides 0401Cit, 0401Arg, 0722Cit, 0722Arg, CCPlus and ArgPlus**. Comparison of the levels of antibodies against each of the peptides in sera from 33 TB patients before and after treatment.

Patients were treated with anti-tuberculous drugs to eradicate the mycobacteria and the reactivity with the (citrullinated) peptides was assessed six months after starting treatment. Serum samples from 33 patients were available for these analyses. The mean levels of antibodies to 0401Cit, 0401Arg, 0722Cit and 0722Arg significantly decreased after treatment (Table 2, Figure 3). Almost all of the patients who were anti-0401Cit and anti-0722Cit positive turned negative after treatment. Eighteen patients who were anti-0401Arg positive and 13 who were anti-0722Arg positive turned negative after treatment. Most of the remaining patients that were reactive with the Arg peptides before treatment (4 and 3 sera, respectively) showed reduced levels of reactivity after treatment. Although the reactivity of this group of patients with the CCPlus peptides was relatively low and, as a consequence, not much difference was observed before and after treatment, the recognition of the ArgPlus peptides was also clearly diminished after treatment.

**Table 2:** Levels of antibodies to citrullinated and corresponding arginine-containing peptides in 33 TB patients before and six months after treatment

Antibodies to	Baseline	After treatment	*P*
**0401Cit**	0.17 ± 0.13	0.10 ± 0.03	*P *< 0.001
**0401Arg**	0.23 ± 0.15	0.12 ± 0.05	*P *< 0.001
**0722Cit**	0.23 ± 0.23	0.13 ± 0.05	*P *< 0.001
**0722Arg**	0.32 ± 0.21	0.17 ± 0.06	*P *< 0.001

### Correlation between the presence of anti-0401Cit/Arg and anti-0722Cit/Arg and clinical characteristics of the patients

The results in Table 3 demonstrate that a significant correlation was found between the presence of anti-0401Cit/Arg and anti-0722Cit/Arg and HIV positivity (*P *< 0.001). Extra-pulmonary involvement as well as African origin correlated with the presence of anti-0722Cit (*P *= 0.024) (Table 3).

**Table 3:** Correlations between clinical data and antibodies to citrullinated and corresponding arginine-containing peptides in patients with mycobacterial infections

Antibodies to	HIV	Use of corticosteroids	Miliary TB	Extra Pulmonary	Africans
**0401Cit**	R = 0.37	R = 0.45	R = 0.19	NS	NS
	*P *< 0.001	*P *< 0.001	*P *= 0.04		
**0401Arg**	R = 0.21	NS	R = 0.18	R = 0.32	R = 0.25
	*P *= 0.013		*P *= 0.06	*P *= 0.000	*P *= 0.006
**0722Cit**	R = 0.41	R = 0.26	R = 0.24	R = 0.37	R = 0.24
	*P *= 0.000	*P *= 0.004	*P *= 0.01	*P *< 0.001	*P *= 0.01
**0722Arg**	R = 0.28	NS	NS	NS	NS
	*P *= 0.001				

Factors such as age, gender, length of symptoms, persistence of positive cultures, NTM, hepatitis C, hepatitis B, multi-drug resistance TB and pulmonary cavitations did not correlate with anti anti-0401Cit/Arg and anti-0722Cit/Arg antibodies.

## Discussion

The present study confirms our previous observation that ACPA are relatively frequently found in the serum of patients with mycobacterial infections. A total of 10 to 16% of patients with mycobacterial infections demonstrated antibodies against citrullinated peptides. However, almost all of these patients also developed reactivity against the non-citrullinated peptides. Interestingly, the frequency by which antibodies to the non-citrullininated peptides were detected was much higher than that of the antibodies reactive with the citrullinated peptides. These results strongly suggest that the antibodies produced by TB and NTM patients are reactive with at least one epitope in the citrullinated peptides, for which the citrulline residue is not critically important. Instead, the presence of an arginine at the position of the citrulline in these peptides appeared to enhance the recognition of this epitope by the patient antibodies.

Our results are consistent with those of Kakumanu et al. who reported that up to 37% of TB patients developed anti-CCP1 antibodies, while many of them reacted to the unmodified arginine-containing peptide as well [10]. The proportion of TB patients who were ACPA positive is similar to our previous observation [8] and significantly higher than in the present study. This is probably due to the methods used in previous studies based on commercial kits for ACPA detection.

Interestingly, we could show that the levels of anti-citrullinated and anti-arginine-containing peptide antibodies significantly decreased after anti-TB treatment. This further supports the hypothesis that the presence of ACPA, like other autoantibodies that have been detected in TB patients, probably reflects a polyclonal non-specific activation of the prolonged inflammatory process [9].

A significant correlation was found between the anti-0401Cit/Arg and anti-0722Cit/Arg antibodies and the presence of HIV. Humoral immunological abnormalities are frequent in HIV and include cryoglobulins, Rheumatoid Factor, antinuclear antibodies, antiphospholipid antibodies and ANCA in 17 to 51% of patients [13,14]. Because the number of HIV infected patients in our study was limited and each of these patients was suffering from TB, further studies are required to investigate the frequency of ACPA and their specificity in patients with HIV.

For two of the patients citrulline-specific reactivities were observed. One of these patients, for which no *after treatment *sample was available, was strongly reactive with the 0401Cit and 0722Cit, as well as the CCPlus peptides, but was not or was only very weakly reactive with the 0401Arg, 0722Arg and ArgPlus peptides, respectively. For the other patient, only the *after treatment *sample displayed strong reactivity with 0722Cit and CCPlus, whereas none of the other peptides was recognized. Both of these patients did not show signs of arthritis, but it might be worthwhile to follow these patients to monitor whether they will develop arthritis and whether they remain ACPA positive.

Previously, a similar citrulline-independent ACPA response has been reported in type 1 autoimmune hepatitis, AIH-1 [7]. However, in case of AIH-1 still a significant number of patients showed citrulline-specific or citrulline-preferred reactivities. In TB the reverse situation is observed, because these patients show arginine-specific or arginine-preferred reactivity.

## Conclusions

In conclusion, ACPA are significantly increased in patients with untreated TB, but in contrast to RA this response is not citrulline-dependent. The results of these studies indicate that ACPA detection in at least a subset of infectious and other inflammatory diseases should be performed by using a combination of citrullinated and the corresponding non-citrullinated antigens.

## Abbreviations

ACPA: anti-citrullinated protein antibodies; AIH-1: type 1 autoimmune hepatitis; Arg: arginine; CCP: cyclic citrullinated peptide; Cit: citrulline; NTM: nontuberculous mycobacterium; PAD: peptidylarginine deiminase; RA: rheumatoid arthritis; RF: rheumatoid factor; TB: tuberculosis.

## Competing interests

The authors declare that they have no competing interests.

## Authors' contributions

OE was responsible for initiative, writing of the protocol and Helsinki approval, statistical analysis and writing of the manuscript. RS and DB recruited patients and sera. RvU was responsible for anti-CCP2 and corresponding arginine control peptide analyses. CO gave technical assistance for ELISA analyses. GP supervised the study, interpreted data, and wrote the manuscript.
